# Multi-Omics Mechanisms of Trimethylamine Oxide and Cardiovascular Disease: A Review

**DOI:** 10.31083/RCM44420

**Published:** 2026-02-04

**Authors:** Huan Yu, Fan Li

**Affiliations:** ^1^Department of General Medicine, Sinopharm Dongfeng General Hospital, Hubei University of Medicine, 442000 Shiyan, Hubei, China

**Keywords:** gut microbes, trimethylamine N-oxide, cardiovascular disease, multi-omics mechanism

## Abstract

Cardiovascular diseases (CVDs) rank among the most prevalent conditions globally, encompassing coronary heart disease, hypertension, cardiomyopathy, and heart failure. The global prevalence of CVD continues to rise despite available therapies such as interventional procedures and pharmacotherapy, which remain associated with high rates of recurrence and mortality. In recent years, with a deepening understanding of the human gut microbiome, researchers have discovered that gut microbiota and their metabolites play a significant role in the development and progression of cardiovascular diseases. Among these, trimethylamine N-oxide (TMAO), a major metabolite of gut microbiota, has garnered extensive attention. Thus, this review leverages a multi-omics perspective to compare the commonalities and differences in TMAO-related mechanisms across various cardiovascular diseases. Moreover, this review aims to construct a TMAO-driven pathogenic network and critically evaluate the translational potential of this metabolite as a disease biomarker and therapeutic target, alongside current challenges.

## 1. Introduction

Cardiovascular diseases (CVDs) constitute a clinical syndrome involving dysfunction of the cardiac and vascular systems, encompassing conditions such as heart failure, hypertension, coronary heart disease, cardiomyopathy, heart valve disease, and arrhythmia [1]. CVD has now become a global health problem [2, 3], posing a significant economic and public health burden while also endangering people’s lives and health. According to the 2023 report released by World Health Organization (WHO) [4], CVD remains the leading cause of death worldwide, resulting in approximately 17.9 million deaths annually, accounting for 32% of global deaths, imposing a substantial disease burden. CVD mortality has long ranked first globally [5].

The incidence, morbidity, and mortality of CVD are still high despite the growing sophistication of current treatments. Recent investigations into the pathophysiology of CVD have revealed that gut flora and its metabolites are also crucial for the development and incidence of the disease [6, 7, 8, 9, 10, 11, 12]. Trimethylamine N-oxide (TMAO) is a major metabolite produced by gut microbiota [13]. High plasma levels of TMAO have been linked in studies to an elevated risk of CVD [14, 15]. A meta-analysis involving nearly 49,000 participants confirmed a significant association between high plasma TMAO levels and the risk of major adverse cardiovascular events (a 41% increase) [16].

To address gaps in existing reviews regarding the breadth of disease coverage and mechanistic depth, this article systematically constructs TMAO’s cardiovascular pathogenic network from a multi-omics integration perspective. We specifically focus on evaluating contradictions between basic research, clinical observations, and Mendelian randomization evidence. Building upon this, we propose a future direction centered on “interventional multi-omics studies”, aiming to provide the field with a new, critical, and comprehensive perspective.

## 2. Biological Characteristics, Generation, Metabolism, and Molecular-Cellular Mechanisms of Trimethylamine N-oxide

TMAO is an oxidized derivative of trimethylamine, a naturally occurring small-molecule amine compound and a metabolic product of the gut microbiota [17, 18]. TMAO is an oxidized derivative of trimethylamine. The primary dietary sources of TMAO in humans include red meat, deep-sea fish, and dairy products, all of which are high in choline, L-carnitine, and betaine, which are necessary building blocks for the production of TMAO [19, 20]. Epidemiological studies have confirmed a significant positive correlation between elevated plasma TMAO levels and the consumption of red meat, deep-sea fish, and dairy products [21]. Following human ingestion, these substances undergo conversion to trimethylamine (TMA) through the action of TMA hydratase enzymes, which is facilitated by gut microbiota, including Firmicutes and Proteobacteria [22, 23, 24]. The TMA then enters the liver via the portal vein circulation. In the liver, flavin monooxygenase 3 (*FMO3*) catalyzes the conversion of TMA into the metabolic byproduct TMAO [25, 26, 27]. Ultimately, most TMAO is excreted via the kidneys in urine [28, 29, 30, 31], with a small portion eliminated through respiration and feces [32].

TMAO participates in various critical biological functions, including osmotic pressure regulation, protein stabilization, and cell division [33]. In 2011, a research team from a cardiovascular disease medical center first reported in Nature that the gut microbiota metabolite TMAO may contribute to heart disease, stimulating investigations into TMAO’s role in cardiovascular and other diseases. Measuring TMAO and its related metabolites provides crucial evidence for preventing and diagnosing these diseases, and related research is gaining increasing attention. Its specific mechanisms include inducing cellular stress, interfering with energy metabolism, and activating inflammatory signaling. We will elaborate on these mechanisms in detail in subsequent chapters, focusing on specific cardiovascular diseases.

## 3. TMAO and Coronary Heart Disease

Coronary atherosclerotic heart disease (CAD), also known as ischemic heart disease, constitutes a significant component of cardiovascular diseases. Its primary characteristic is narrowing or obstruction of the vascular lumen caused by atherosclerosis (AS), leading to myocardial ischemia and hypoxia. Metabolomics and metagenomics studies indicate a strong correlation between the formation of atherosclerotic plaques and plasma TMAO levels [34, 35]. TMAO serves as an independent predictor and promoter of AS [36, 37, 38, 39]. Its underlying mechanisms involve multiple omics, summarized as follows: (Table 1, Ref. [40, 41, 42, 43, 44, 45, 46]).

**Table 1.** **Basic studies on the association of TMAO with the development of atherosclerosis**.

Experimental subject	Experimental method	Outcome	Conclusion	Discussion	References
*ApoE-/-* mice	Eight male mice were in each of the experimental and control groups. One group was fed a diet containing 0.3% TMAO for 8 weeks (n = 8)	Expression of cholesterol 7α-hydroxylase (*Cyp7a1*) in the group of TMAO-intervened mice was 38.4% lower than that of mice on the control diet (*p * < 0.05)	Inhibition of hepatic bile acid synthase Cyp7a1 by TMAO leads to disruption of bile acid-related pathways and promotes atherosclerosis	TMAO-induced suppression of hepatic bile acid synthase Cyp7a1 expression disrupted bile acid-related pathways and promoted atherosclerosis. Using a classical atherosclerosis model, the correlation is strong; however, the results demonstrate correlation rather than direct causation.	[40]
TMAO-induced HUVEC, vascular smooth muscle cells	Both cell types were pretreated with 100 nmol/L NF-κB activation inhibitor IV. One group was then treated with 200 µmol/L TMAO overnight (n = 6)	TMAO-induced expression of inflammatory genes was suppressed in both cells (*p * < 0.01)	NF-κB signalling is required for TMAO-induced inflammatory gene expression in endothelial and smooth muscle cells	Time-limited: Most experiments observed responses within 6 hours only, without evaluating the effects of prolonged TMAO exposure.	[41]
CAEC	Cells were treated with TMAO (30 µm) (n = 5–6)	TMAO treatment increases co-localisation of NLRP3 with ASC in CAECs	NLRP3 inflammatory vesicles are formed and activated in TMAO-treated arterial endothelial cells	Cell type homogeneity: Only mouse CAECs were used; no validation was performed on human-derived or different vascular bed endothelial cells.	[42]
HUVEC *ApoE-/-* mice	1. Induced by different concentrations of TMAO	1. Intracellular ROS were significantly higher in the TMAO-stimulated group than in the control group (*p * < 0.05)	TMAO triggers ROS and activates TXNIP-NLRP3 inflammatory vesicles	This is a purely *in vitro* cellular study. Whether TMAO functions through the same mechanism in the complex systemic environment of animals or humans cannot be verified.	[43]
	2. ROS inhibitor NAC treatment (n = 5)	2. NAC treatment or siRNA-mediated knockdown of TXPIN and NLRP3 reverses TMAO-mediated inflammation			
HUVECs	1. Endothelial cells treated with different concentrations of TMAO	1. TMAO exposure reduced SOD2 activity and SIRT3 expression (*p * < 0.05)	The SIRT3-SOD2 pathway is required for the induction of mtROS accumulation by TMAO in endothelial cells and subsequent activation of NLRP3 inflammatory vesicles	The demonstration of the entire signaling pathway “SIRT3↓ → SOD2 activity↓ → mtROS↑ → NLRP3 inflammasome activation” is more correlational than strictly causal.	[44]
	2. SIRT3 siRNA transfection (n = 3)	2. Elevated levels of total ROS and mtROS and reduced SOD2 activity (*p * < 0.01)			
*ApoE-/-* mice	Ten mice per group (experimental and control). The experimental group was fed 1 mmol/L TMAO (n = 6)	1. TMAO enhanced macrophage recruitment, CD36, and pro-inflammatory cytokine expression in plaque lesions	The CD36/MAPK/JNK pathway plays a key role in TMAO-induced foam cell formation	Although *in vitro* experiments using MAPK/JNK inhibitors demonstrated suppressed CD36 expression and foam cell formation, these findings remain unvalidated in animal models (e.g., inhibitor treatment or knockout mice).	[45]
		2. Both MAPK inhibitors and JNK inhibitors can reduce ox-LDL and TMAO-induced CD36 expression			
Washed platelet	One group exposed to TMAO (n = 4)	Calcium levels were significantly higher in the TMAO-exposed group	TMAO promotes intracellular calcium ion release	*In vitro* studies confirm TMAO’s effects on platelet function; however, the washed platelet model simplifies the complex *in vivo* blood environment; its prothrombotic capacity requires validation in hemodynamic models.	[46]

HUVEC, human umbilical vein endothelial cells; *ApoE-/-* mice, ApoE knockout mice; CAEC, mouse carotid artery endothelial cells; RCT, reverse cholesterol transport; ROS, reactive oxygen species; NAC, N-acetylcysteine; mtROS, mitochondrial reactive oxygen species; TXNIP, thioredoxin interacting protein; SOD2, superoxide dismutase 2; SIRT3, mitochondrial sirtuin 3; NLRP3, NOD-like receptor heat protein structural domain-related protein 3; MAPK, mitogen-activated protein kinase; JNK, c-Jun amino-terminal kinase; TMAO, trimethylamine N-oxide.

TMAO influences cholesterol metabolism [47]. TMAO primarily accelerates atherosclerosis by promoting cholesterol influx and blocking the bile acid pathway [48, 49]. Metabolomics studies revealed that, compared to normal mice, mice fed a TMAO-supplemented diet exhibited a 35% reduction in reverse cholesterol transport (RCT) capacity and significantly elevated plasma cholesterol levels. In contrast, cholesterol levels in the liver and bile did not show any significant changes. This finding suggests TMAO influences cholesterol metabolism by affecting RCT. Furthermore, transcriptomic analysis revealed that TMAO-mediated transcriptional regulation suppressed mRNA expression of key hepatic bile acid synthesis enzymes (CYP7A1, CYP27A1). Subsequent proteomic studies demonstrated that post-translational regulation downregulated the abundance of bile acid transporters, leading to disrupted bile acid pathways and cholesterol accumulation, which promotes atherosclerosis [50].

TMAO promotes vascular inflammation and endothelial dysfunction [42]. Inflammation permeates the entire process of AS and related cardiovascular events [51]. As a key driver of AS progression, TMAO induces vascular inflammation and endothelial dysfunction through dual mechanisms ((nuclear factor kappa-light-chain-enhancer of activated B cells) NF-κB and (NOD-like receptor family pyrin domain containing 3) NLRP3 pathways). Proteomics studies confirm that TMAO activates the mitogen-activated protein kinase (MAPK) pathway via multiple kinases, promoting (inhibitor of κB alpha) IκBα phosphorylation and thereby driving the NF-κB pathway [52], which leads to vascular inflammation and endothelial dysfunction. On the other hand, metabolomics revealed that TMAO induces mitochondrial dysfunction, while proteomics showed suppressed expression of the deacetylase mitochondrial sirtuin 3 (SIRT3), leading to mtROS accumulation. Proteomics further detected mitochondrial reactive oxygen species (mtROS) activation of NLRP3 inflammasome components and Caspase-1 activation [44].

TMAO promotes macrophage foam cell formation [53, 54]. Oxidized low-density lipoprotein (ox-LDL) internalization by macrophages is a crucial step in driving lipid accumulation and the transformation of macrophages into foam cells [55]. In proteomics studies investigating TMAO’s atherogenic effects, researchers found that TMAO significantly upregulates the expression levels of macrophage scavenger receptors Class A Scavenger Receptor Type I (SR-A1) and Cluster of Differentiation 36 (CD36) [56]. Transcriptomic analysis further revealed that this process may be mediated by the MAPK/c-Jun amino-terminal kinase (JNK) pathway [45]. Receptor upregulation enhances macrophage phagocytosis of ox-LDL, while metabolomic studies reveal substantial intracellular lipid accumulation, ultimately leading to the formation of foam cells—the pathological basis of atherosclerotic plaques [57].

TMAO has been demonstrated to regulate platelet activation and aggregation, thereby influencing thrombotic risk. Platelets play a critical role in maintaining the pro- and anti-thrombotic balance and blood flow homeostasis. Several animal model studies found that acute elevations of circulating TMAO enhance the potential for thrombosis *in vivo* [58, 59]. Functional proteomics experiments revealed that TMAO triggers Inositol 1,4,5-trisphosphate (IP3)-dependent calcium ion (Ca^2+^) release within platelets, activates Protein Kinase C (PKC) and calcineurin, and induces granule secretion. Activated platelets release multiple growth factors (e.g., Platelet-Derived Growth Factor (PDGF), Vascular Endothelial Growth Factor (VEGF)), which in turn activate the MAPK/extracellular signal-regulated kinase (ERK) pathway in vascular smooth muscle cells, driving their proliferation and migration to contribute to plaque progression [60, 61, 62]. This mechanism was validated *in vitro* models. When washed human or mouse platelets were exposed to pathologically relevant concentrations of TMAO, significantly enhanced responsiveness to classical agonists (e.g., collagen, Adenosine Diphosphate (ADP)) was observed, characterized by accelerated aggregation kinetics and heightened aggregation levels [46].

## 4. TMAO and Hypertension

Hypertension is a clinical syndrome primarily characterized by elevated blood pressure in the systemic arteries. Additionally, hypertension is often accompanied by functional or structural damage to organs such as the heart, brain, and kidneys. A study examining the relationship between TMAO levels and hypertension risk in CVD patients found that compared to CVD patients with low TMAO concentrations, those with high TMAO concentrations had a 14% increased risk of hypertension. Furthermore, a linear dose-response relationship exists between circulating TMAO concentrations and hypertension risk in CVD patients. Each 1 µmol/L increase in circulating TMAO concentration is associated with a 1.014% increase in hypertension risk. These findings suggest a direct link between circulating TMAO levels and heightened hypertension risk in CVD patients [63]. Multi-omics studies indicate that TMAO contributes to hypertension pathogenesis through the following mechanisms.

TMAO induces sympathetic nervous system excitation. Studies have revealed that rats on a high-salt diet exhibit significantly elevated TMAO levels in plasma and cerebrospinal fluid, higher blood pressure, and more pronounced sympathetic nervous system activity. Multi-omics analysis revealed the following sequence of events: Metabolomics detected high levels of TMAO; Transcriptomics identified upregulation of gene expression associated with neuro-oxidative stress and sympathetic nervous excitation in brain regions; Proteomics revealed activation of related proteins, ultimately leading to elevated blood pressure [64].

TMAO enhances vascular sensitivity to Angiotensin II (Ang II) [65]. Although no studies have directly demonstrated TMAO’s impact on blood pressure, it potentiates the hemodynamic effects of angiotensin. Proteomics studies reveal TMAO’s dual-pathway amplification effect. High-concentration TMAO directly constricts blood vessels to elevate blood pressure [66], whereas low-concentration TMAO activates the endoplasmic reticulum stress kinase PKR-like endoplasmic reticulum kinase (PERK), triggering the ROS/Ca^2+^/calmodulin-dependent protein kinase II (CaMKII) cascade, phosphorylating Phospholipase C β3 (PLCβ3), and promoting calcium release, ultimately enhancing Ang II-induced vasoconstriction [67].

TMAO activates the Arginine Vasopressin- Aquaporin 2 (AVP-AQP2) axis to promote blood pressure elevation. Metabolomics studies in spontaneously hypertensive rats (SHR) revealed a positive correlation between plasma TMAO levels and plasma osmolarity. Transcriptomics and Proteomics confirmed that this triggers regulation of the arginine vasopressin (AVP)-aquaporin 2 (AQP-2) axis, promoting water reabsorption, increasing blood volume, and thereby elevating blood pressure [68].

Furthermore, elevated TMAO may be a novel mechanism contributing to aortic stiffness. Blood pressure rises when the aorta stiffens because its elasticity declines and blood flow velocity increases [69]. Proteomics studies indicate that TMAO-induced accumulation of advanced glycation end-products (AGEs) and superoxide-related oxidative stress collectively contribute to aortic stiffening and elevated systolic blood pressure [70, 71], ultimately triggering hypertension.

Currently, the majority of research on the connection between TMAO and hypertension has been conducted in animal models. Future population-based intervention trials and organ-like models are required to confirm the pathophysiological processes of this association.

## 5. TMAO and Arrhythmias

An irregularity in the source of cardiac impulses, the frequency and rhythm of heartbeats, and the conduction of impulses is known as an arrhythmia. Current research on TMAO and arrhythmia focuses on its association with atrial fibrillation (AF), a common arrhythmia in the population, with an incidence of 1–2% [72]. Research has indicated that TMAO can exacerbate the atria’s electrical instability [73]. Its mechanisms involve multiple pathways.

TMAO activates the cardiac autonomic nervous system (CANS) [74]. Research on TMAO and CANS indicates that stimulating atrial autonomic nerves can induce electrical remodeling and promote the development of paroxysmal AF [75]. Proteomics studies reveal that TMAO activates the nervous system either directly through specific protein receptor ion channels [76] or indirectly by stimulating nerves via inflammatory signaling pathways [77]. Transcriptomics further indicates that TMAO induces gene expression reprogramming within the nucleus, leading to “electrical remodeling” and arrhythmias.

TMAO Induces myocardial hypertrophy. Clinical observations indicate that AF and various other arrhythmias commonly occur in patients with cardiac hypertrophy and fibrosis [78]. Proteomics studies reveal that TMAO activates intracellular transforming growth factor beta 1 (TGF-β1)/SMAD family member 3 (Smad3) and PERK/inositol-requiring enzyme 1 alpha (IRE1α) signaling pathways [79]. These protein events ultimately converge in the nucleus, followed by transcriptomic-driven gene expression reprogramming. These gene expression alterations lead to cardiomyocyte hypertrophy and interstitial fibrosis—collectively termed “cardiac remodeling”. The resulting structural changes in hypertrophied and fibrotic hearts create heterogeneous electrical conduction patterns, facilitating the formation of reentrant circuits and indirectly contributing to arrhythmias. Further animal studies confirm that elevated TMAO levels directly induce myocardial hypertrophy and fibrosis, while intervention with the Smad3 inhibitor specific inhibitor of Smad3 (SIS3) attenuates these processes [80]. This evidence demonstrates TMAO’s role in inducing cardiac hypertrophy via the TGF-β1/Smad3 pathway.

TMAO promotes inflammation [81, 82], causing vascular endothelial dysfunction. Inflammation plays a pivotal role in the onset and progression of AF. The NLRP3 inflammasome in inflammatory responses not only induces atrial structural remodeling but also shortens atrial action potential duration, promotes reentry, increases intracellular Ca^2+^ release, and induces abnormal electrical activities such as delayed afterdepolarization [83], ultimately leading to electrophysiological remodeling and promoting AF occurrence. Proteomics studies indicate TMAO primarily activates NLRP3 inflammasomes through multiple pro-inflammatory signaling pathways (e.g., NF-κB activation or SIRT3-superoxide dismutase 2 (SOD2)-mtROS signaling) [84]. Transcriptomics reveal NLRP3 inflammasome-induced inflammatory gene expression causes vascular endothelial injury [85], thereby triggering arrhythmias [86].

TMAO promotes vascular aging and endothelial dysfunction. Vascular structural and functional impairments contribute to AF through multiple pathways. Proteomics and transcriptomics studies synergistically revealed that TMAO mediates suppression of silencing regulatory protein 1 (SIRT1) expression [87], subsequently activating the tumor suppressor protein (p53)/cell cycle-dependent kinase inhibitor (p21)/retinoblastoma protein (Rb) pathway. This leads to increased acetylation of p53 and p21, as well as reduced phosphorylation levels of cyclin-dependent kinase 2 (CDK2) and Rb, ultimately inducing endothelial cell senescence and arterial aging [88]. This illustrates an additional mechanism through which TMAO contributes to cardiovascular diseases, including arrhythmia.

Controversy and future perspectives on the association between TMAO and atrial fibrillation.

Despite substantial basic research indicating an association between TMAO and an increased risk of AF, its causal relationship warrants cautious interpretation, and significant controversy persists in this field.

A clinical study involving 45 AF patients and 20 non-AF individuals found that TMAO levels in AF patients were comparable to those in non-AF individuals (*p* = 0.629). No association was observed between TMAO and the AF progression phenotype (*p* = 0.588). Among the 45 AF patients, TMAO was remeasured 12–18 months after transcatheter ablation for AF. TMAO levels were comparable at baseline and follow-up (r = 0.481, *p* = 0.003), indicating that increased TMAO levels were unrelated to ablation success (restoration of sinus rhythm) [89]. Data from this pilot study suggest that TMAO levels are not universally elevated in atrial fibrillation and are unrelated to the progression phenotype of AF. More importantly, recent studies utilizing Mendelian randomization (MR) have not provided strong support for a causal relationship between TMAO and AF. A large-scale MR analysis similarly found no significant causal association between genetically elevated TMAO levels and AF risk (*p* = 0.961) [90]. This discrepancy suggests that the observed correlation between TMAO and AF in observational studies may be confounded by unknown factors or reverse causality (i.e., AF leading to gut dysfunction and elevated TMAO). TMAO may not be a direct driver of AF onset but rather a mediator of upstream factors (e.g., poor diet, gut dysbiosis) or downstream consequences (e.g., impaired cardiac and renal function). This suggests TMAO acts as a “promoting” factor rather than a “root cause”.

The inconsistencies in the aforementioned research findings may be attributed to multiple factors: (1) Sample size and population heterogeneity: Most positive studies have large sample sizes, whereas negative results may be constrained by small-sample effects. (2) Differences in patient baseline characteristics (e.g., age, renal function, comorbidities, dietary patterns) may confound the true association between TMAO and AF. (3) AF subtype and disease duration: TMAO may play a more significant role in paroxysmal AF than in persistent AF, or its effect may be confined to other specific AF subtypes. This hypothesis requires validation through more refined clinical stratification studies.

## 6. TMAO and Cardiomyopathy

Hypertrophic cardiomyopathy (HCM) is a structural myocardial disorder characterized by ventricular hypertrophy/dilatation, accompanied by mechanical dysfunction and electrical abnormalities, ultimately leading to malignant arrhythmias and heart failure. Recent multi-omics studies have revealed that TMAO, a gut microbiota metabolite, contributes to the pathophysiology of cardiomyopathy through several mechanisms.

TMAO induces myocardial fibrosis. Multi-omics studies indicate that TMAO induces myocardial fibrosis by activating multiple parallel pro-fibrotic signaling pathways. Proteomics evidence shows that TMAO significantly activates TGF-β1/Smad3 [91], JAK2/STAT3 [92], and TGF-βRI/Smad2 pathways. Subsequent transcriptomic analysis confirmed that these activated transcription factors subsequently initiate programmatic transcription of pro-fibrotic genes within the nucleus, ultimately leading to upregulation of fibronectin, collagen I/III, and alpha-smooth muscle actin (α-SMA) expression, collectively promoting myocardial fibrosis [93]. Regarding myocardial remodeling, proteomic studies on hypertrophic cardiomyopathy revealed that TMAO enhances protein kinase A (PKA) activity within myocardial tissue, inducing specific phosphorylation of ryanodine receptor 2 (RyR2) at the tryptophan 2808 site [94]. This process has been shown to trigger ventricular remodeling and lead to a significant impairment of cardiac contractile function.

TMAO-induced mitochondrial energy metabolism dysregulation [95]. Proteomics studies reveal that TMAO inhibits the function of key mitochondrial enzymes and protein complexes. Metabolomics detects reduced intracellular ATP synthesis, with the accumulation of metabolic waste and energy depletion directly causing myocardial tissue damage [96].

Notably, TMAO research in cardiomyopathy remains in its infancy compared to the field of atherosclerosis, with evidence primarily derived from animal models and cellular experiments. Current evidence is limited and lacks large-scale epidemiological validation. Furthermore, myocardial fibrosis progresses over extended periods, whereas existing studies predominantly involve acute or subacute interventions. The long-term effects of TMAO remain unclear. Therefore, current perspectives should be regarded as exploratory hypotheses, offering novel insights into these diseases; however, significant progress is still required before clinical application.

## 7. TMAO and Heart Valve Disease

Heart valve disease involves structural abnormalities characterized by valve stenosis or regurgitation. TMAO is recognized to facilitate the onset and advancement of atherosclerosis by eliciting inflammatory and stress responses. Calcific aortic valve disease (CAVD) exhibits analogous risk factors and pathological traits to atherosclerosis. TMAO is speculated to be associated with the progression of aortic valve calcification and rigidity [97, 98]. The association between TMAO and CAVD is supported by research, and the underlying mechanisms are summarized below:

TMAO promotes aortic valve cell calcification via inflammatory responses. Transcriptomic studies reveal that TMAO induces expression of Methyl Transferase-Like 3 (Mettl3) in macrophages. Proteomic analyses indicate Mettl3 suppresses N6-Methyladenosine (m6A)/YTH domain family protein 2 (YTHDF2) pathway to suppress interleukin-1 receptor-associated kinase M (IRAK-M) expression. As IRAK-M is a key negative regulator of the NF-κB signaling pathway [99], NF-κB plays a primary role in TMAO-induced osteogenic responses in human aortic valve interstitial cells (AVICs). Thus, TMAO indirectly induces NF-κB phosphorylation via the Mettl3/IRAK-M pathway, exacerbating inflammatory responses in aortic valve interstitial cells and ultimately worsening aortic valve calcification.

TMAO promotes collagen fibrillation activity via stress responses [100]. Studies reveal that TMAO-treated AVICs exhibit marked fibrosis. Proteomics studies indicate TMAO activates endoplasmic reticulum stress in AVICs, leading to PERK and IRE1α protein activation and modification, producing Activating Transcription Factor 4 (ATF4) and X-box binding protein 1, spliced isoform (XBP-1s) transcription factors. Transcriptomic analysis subsequently revealed that these pathways enhance collagen fibrotic activity by upregulating TGF-β1 and collagen I expression in human AVICs, demonstrating that TMAO’s pro-fibrotic effect on human AVICs is mediated by the PERK/ATF-4 and inositol-requiring enzyme 1 alpha (IRE-1α)/X-box binding protein 1, spliced form (XBP-1s) pathways [101, 102]. TMAO enhances collagen fibrillation activity [103], leading to abnormal collagen deposition and aortic valve thickening, which further induces valve stiffness, stenosis, and calcification.

Current research on the pathophysiological mechanisms linking TMAO to other valvular diseases has not yielded definitive conclusions. Existing findings primarily stem from cellular experiments. Given the complexity of human physiology, it remains uncertain whether these mechanisms translate to human subjects. Furthermore, large-scale clinical validation is lacking. Further exploration is needed to elucidate the mechanisms linking TMAO to valvular diseases.

## 8. TMAO and Heart Failure

Heart failure (HF) is a clinical syndrome characterized by impaired ventricular filling or ejection function due to various structural or functional cardiac diseases, leading to congestion in the pulmonary or systemic circulation and inadequate organ perfusion. It represents the end-stage manifestation of multiple cardiac diseases [104, 105]. Reduced cardiac output and blood redistribution in HF patients decrease intestinal perfusion and compromise the intestinal barrier [106], facilitating the translocation of gut microbiota and endotoxins into the bloodstream [107, 108].

This process exacerbates systemic inflammation and worsens HF. Studies indicate that the intestinal mucosal barrier is damaged in HF patients, with significant alterations in gut microbiota composition and proportion, leading to the production of various metabolites. As one such metabolic product, TMAO is closely associated with HF development [109, 110]. Plasma TMAO concentrations in heart failure patients exhibit a significant increase as the disease progresses and are closely correlated with the severity of the disease [111]. Research suggests that TMAO is not merely a passive biomarker; it is actively involved in the deterioration of HF, hence establishing a detrimental cycle. The following is a summary of the multi-omics mechanisms through which TMAO exacerbates heart failure:

TMAO promotes myocardial hypertrophy and fibrosis. Myocardial hypertrophy and fibrosis are common organic lesions in heart failure [112]. The pathophysiology associated with TMAO has been thoroughly described in the preceding chapter on cardiomyopathy and will not be repeated here.

TMAO aggravates heart failure via inflammatory reactions. Inflammation is a key driver of heart failure progression and poor outcomes. Localized and systemic inflammation are prominent features in the progression of chronic heart failure [113]. Further proteomics studies revealed that TMAO promotes mtROS accumulation by inhibiting SIRT3 and SOD2 activity, thereby activating the NLRP3 inflammasome [44]. This inflammasome secretes Caspase-1, interleukin (IL)-1β, and IL-18, inducing vascular inflammation and further exacerbating heart failure.

TMAO impacts heart energy metabolism. After administering mice TMAO for eight weeks, Makrecka-Kuka *et al*. [96] found that prolonged TMAO exposure induces severe cardiac energy metabolism disorders. Proteomics analysis indicated this occurs via suppression of pyruvate dehydrogenase complex (PDH) function and impairment of fatty acid β-oxidation, while metabolomics directly detected reduced energy substrate flux and insufficient ATP production. This energy-depleted state further triggers more severe pathological alterations. Subsequent proteomics studies confirmed that TMAO induces damage to the T-tubule network and disrupts calcium handling [114, 115], ultimately contributing to ventricular remodeling and heart failure.

TMAO-induced renal tubulointerstitial fibrosis and dysfunction [116, 117]. During heart failure progression, renal function alterations prove equally critical alongside cardiac injury [118]. Heart failure induces intestinal congestion and dysbiosis, with metagenomic analyses detecting elevated TMAO production. Excessive TMAO excretion through the kidneys is thought to accelerate the deterioration of renal function [119]. TMAO directly exerts toxicity on renal tubular cells and induces renal interstitial fibrosis (via proteomic/transcriptomic mechanisms). Once TMAO begins impairing renal function, the kidneys’ capacity to clear TMAO diminishes, leading to further accumulation of TMAO in the bloodstream and forming a metabolic positive feedback loop at the omics level [120]. Renal insufficiency exacerbates cardiac burden through water and sodium retention and toxin accumulation. This accumulation, in turn, exacerbates heart failure [121]. This pathogenic mechanism was validated in intervention studies: in a rat model of chronic cardio-renal syndrome, treatment with the TMA formation inhibitor 3,3-dimethyl-1-butanol (DMB) to reduce TMAO levels improved both cardiac and renal function [122, 123]. The findings above provide sufficient evidence that TMAO can exacerbate the decline in renal function, and Hu *et al*. [124] directly demonstrated that reducing TMAO levels effectively delays renal fibrosis progression. These interventional findings establish TMAO’s pivotal role in this pathological process through both positive and negative effects.

However, despite multi-omics evidence pointing to TMAO exacerbating heart failure via the cardio-renal axis, its clinical role remains mired in a “causal dilemma”. This study and most observational research confirm TMAO as a potent prognostic predictor of HF [125, 126]. Nevertheless, the central controversy lies in whether its elevation drives heart failure progression or arises as a consequence of heart failure-induced intestinal congestion, barrier disruption, and renal decline. This potential reverse causality and renal function confounding complicate establishing TMAO’s direct effects in clinical studies. Further complicating matters, some studies report that moderate increases in plasma TMAO levels do not adversely affect the circulatory system. Increasing dietary TMAO intake alleviates diastolic dysfunction in stress-overloaded rat hearts [127, 128]. This apparent bidirectional effect further complicates the mechanism, suggesting its action may be dose- and pathology-state dependent. However, we must cautiously note that this compelling hypothesis currently rests on very preliminary evidence. First, this low-dose protective effect has been reported in only a handful of studies to date and lacks independent replication in humans. Second and more critically, the precise plasma concentration “threshold” distinguishing its potential beneficial from harmful effects has not been systematically defined in any species. Consequently, significant uncertainty remains regarding the universality of this conclusion and its generalizability to the entire population.

## 9. Challenges and Prospects of TMAO as a Clinical Biomarker

Although basic research strongly suggests TMAO is a pathogenic factor in cardiovascular disease, its journey toward becoming a clinical biomarker requires careful evaluation. This section compares TMAO with existing biomarkers in terms of diagnostic performance and clinical feasibility, and explores its unique translational value.

### 9.1 Diagnostic Performance: Sensitivity, Specificity, and Predictive Value

Comparison with high-sensitivity C-reactive protein (hs-CRP): hs-CRP serves as the gold standard for measuring systemic inflammation, offering mature testing protocols and low costs. However, regarding disease specificity, TMAO shares similarities with hs-CRP: elevated levels occur across multiple conditions rather than being unique to cardiovascular disease, limiting its specificity as a standalone diagnostic marker.

Compared to B-type natriuretic peptide (BNP), BNP is a cornerstone for diagnosing and managing HF, exhibiting high sensitivity and specificity by directly reflecting ventricular wall tension. TMAO similarly demonstrates robust prognostic predictive value in HF, but its mechanism is more focused on reflecting systemic metabolic disorders and cardio-renal interactions. Therefore, TMAO can complement BNP. BNP indicates heart stress, while TMAO indicates the cause of cardiac deterioration.

###  9.2 Clinical Feasibility and Standardization Challenges

Currently, the most significant barrier to TMAO testing entering clinical practice is feasibility. BNP and hs-CRP testing are already automated, standardized, and cost-effective. Precise measurement of plasma TMAO concentrations typically requires liquid chromatography-tandem mass spectrometry. This technique is complex to operate, costly, and not yet standardized across clinical laboratories. Therefore, developing rapid, low-cost, automated testing methods is a prerequisite for its clinical translation.

## 10. Conclusions

Cardiovascular disease management and treatment remain challenging tasks on a global scale. Recent research has demonstrated that intestinal flora and its metabolites—TMAO being one of the primary metabolites of intestinal flora linked to human diseases—play a significant role in the onset and progression of cardiovascular disorders. TMAO has a substantial role in the development of cardiovascular illnesses as a co-pathological factor. Its pro-atherosclerotic role in coronary artery disease is well established. Recent research has demonstrated that TMAO is involved in the development of several cardiovascular diseases, including heart failure, heart valve disease, and cardiomyopathy, by promoting multiple omics mechanisms.

**Fig. 1.**
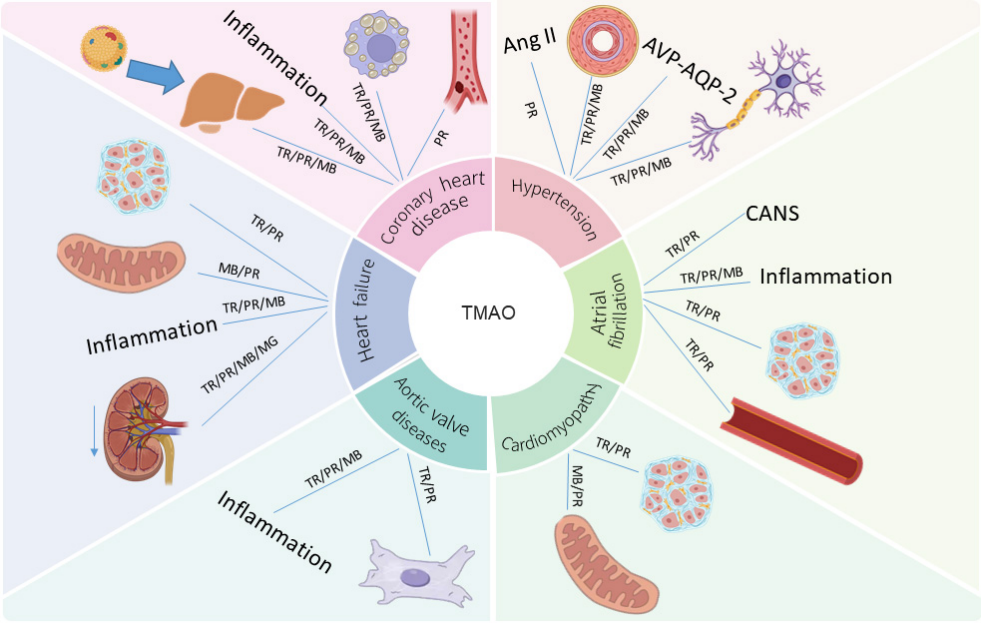
**A multi-omics integrated network of gut microbiota-derived metabolite TMAO driving cardiovascular diseases**. The downward arrow indicates a decline in renal function. TR, Transcriptomics; PR, Proteomics; ME, Metabolomics; MG, Metagenomics. Figure created with BioRender.

This review has synthesized evidence demonstrating TMAO’s involvement in the development of cardiovascular disease through multiple pathways, including interference with cholesterol metabolism, triggering inflammatory responses, promoting fibrosis, and affecting energy homeostasis (Fig. 1). The convergence of multi-omics evidence provides strong support for its pathogenic mechanisms, yet also reveals complex controversies and challenges, such as inconsistencies between observational studies and Mendelian randomization findings, dose-dependent bidirectional effects, and unavoidable confounding factors.

To achieve breakthroughs, future research must transcend simple correlational descriptions and adopt more integrative and causally inferential strategies. Future directions should include: (1) Advancing longitudinal, intervention-based multi-omics studies: The concurrent collection of genomic, metabolomic, and proteomic data from a single cohort for dynamic monitoring, along with integrated bioinformatics analysis across various time points, confirms the causal role of TMAO and identifies key molecular modules that are most responsive to TMAO levels. This facilitates a transition from “associated pathways” to “precision targets”. (2) Deepen tissue-specific mechanism studies: Employ cutting-edge technologies like spatial omics and organoids to precisely knock out or overexpress key TMAO metabolic enzymes (e.g., FMO3) in controlled environments. This approach will directly validate pathogenic mechanisms within specific cell types, circumventing confounding factors inherent in systemic studies. (3) Define clinical translation pathways: Focus on resolving critical issues, including standardization of TMAO detection, cost-benefit assessment, and its complementary value relative to traditional biomarkers (e.g., BNP, hs-CRP). Explore its potential as a biomarker to pave the way for clinical implementation. Ultimately, achieve personalized cardiovascular disease prevention and treatment strategies based on gut microbiota regulation.
